# Mucosa-Associated Lymphoid Tissue Lymphoma of the Lacrimal Gland: Sustained Remission after Eradication of Helicobacter Pylori Infection

**DOI:** 10.1155/2011/945752

**Published:** 2011-12-07

**Authors:** Mohammed Hasosah, Abdullah Baothman, Mohamed Satti, Suzanne Kutbi, Khaled Alghamdi, Kevan Jacobson

**Affiliations:** ^1^Department of Pediatric Gastroenterology, King Saud Bin Abdulaziz University for Health Sciences/National Guard Health Affairs, P.O. Box 8202, Jeddah 21482, Saudi Arabia; ^2^Pediatric Hematology/Oncology Department, King Saud Bin Abdulaziz University for Health Sciences/National Guard Health Affairs, National Guard Hospital-Western Region, Jeddah 21482, Saudi Arabia; ^3^Pathology Department, King Saud Bin Abdulaziz University for Health Sciences/National Guard Health Affairs, National Guard Hospital-Western Region, Jeddah 21482, Saudi Arabia; ^4^Pediatric Department, King Saud Bin Abdulaziz University for Health Sciences/National Guard Health Affairs, National Guard Hospital-Western Region, Jeddah 21482, Saudi Arabia; ^5^Pediatric Gastroenterology Department, British Colombia Children's Hospital, University of British Columbia, Vancouver, BC, Canada V6H 3V4

## Abstract

Mucosa-associated lymphoid tissue (MALT) lymphoma is the third most common non-Hodgkin lymphoma, and it is strongly associated with helicobacter pylori infection of the stomach. MALT lymphoma of the lacrimal gland usually presents as a localized disease process in extranodal tissues. The treatment options of MALT lymphoma of the lacrimal gland chiefly include radiation of the tumor, chemotherapy, surgical removal, or a combination of these strategies. We report a case of localized MALT lymphoma of the lacrimal gland, with prolonged sustained remission after eradication of gastric Helicobacter pylori (H. Pylori) infection. He sustains in remission of lacrimal MALT lymphoma for four years without chemotherapy or radiotherapy.

## 1. Introduction

Mucosa-associated lymphoid tissue (MALT) lymphoma is the third most common non-Hodgkin lymphoma subtype, accounting for around 6–8% of all non-Hodgkin lymphomas [1]. Extranodal marginal zone B-cell lymphoma (EMZBL) also referred to as MALT lymphoma of the lacrimal gland is a rare condition [2]. The stomach is the most frequent site of involvement by EMZBL, followed by salivary glands, orbit, lung, and skin.

*H. pylori* infection of the stomach is recognized as a causative agent of gastritis, ulcer disease, gastric carcinoma and MALT lymphoma [3]. 

The treatment options of MALT lymphoma of the lacrimal gland chiefly include radiation of the tumor, chemotherapy, surgical removal, or a combination of these strategies [4]. 

The present paper describes a 6-year-old boy with MALT lymphoma of the lacrimal gland with sustained remission after eradication of gastric *H. pylori* infection and without chemotherapy or radiotherapy. A review of the literature is also provided.

## 2. Case Report

A 6-year-old boy presented with lacrimal sac swelling for further investigation. The patient was noticed to have a swelling at the left nasal aspect for the last 3 months. It increased gradually in size associated of excessive tearing. There was no history of fever, weight loss, and change in appetite or activity. No history of night sweat, skin rash, joint pain, recent travel. Medical history was otherwise noncontributory. The family revealed no history of malignancy. The patient had normal developmental milestones. He was not taking medications.

On admission to hospital, the physical examination revealed no evidence of growth retardation and a fullness over the left lacrimal sac area; otherwise extraocular motility was full with minimal ptosis. Irrigation of the lacrimal system was negative. Cornea, anterior chamber, and lens were clear on both sides. No hepatosplenomegaly, lymphadenopathy, or skin rashes were noted. Physical systemic examination was otherwise unremarkable.

The laboratory findings revealed normal haemoglobin, white blood cell, and platelet counts. The electrolytes, coagulation study, liver function tests, Lactate dehydrogenase (LDH), and peripheral blood smear results were normal. Cerebrospinal fluid studies revealed no blast cells.

Bone marrow studies (aspirate, biopsy, clot section, and iron stain) showed normocellular active bone marrow with no evidence of infiltration by lymphoma or any other malignancy. 

A computed tomography (CT) scan revealed a soft tissue mass located and extended out of the left lacrimal sac with enhanced posterior encroachment into the orbit. CT scan revealed that bilateral enlarged cervical lymph nodes otherwise the brain, orbit, chest, abdomen, and pelvis were normal. Magnetic resonance imaging (MRI) showed Dacryocystocele. 

**Figure 1:**
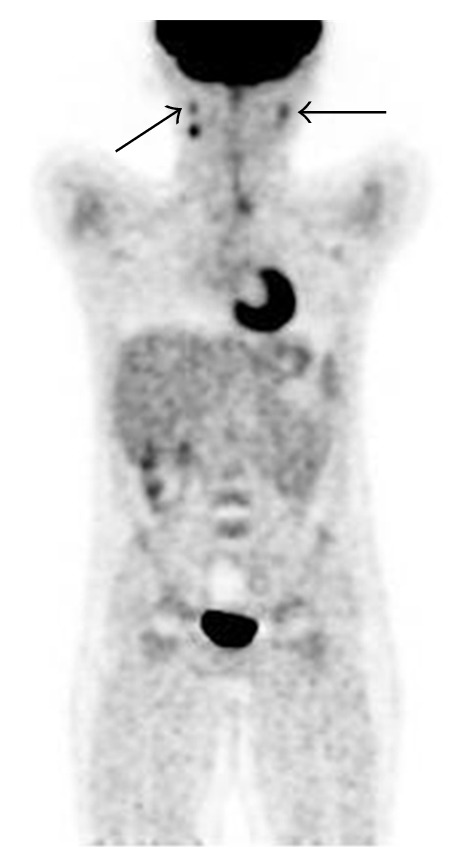
PET-CT of whole body shows two right and one left small hypermetabolic nodal lesions in the upper jugulodigastric area (black arrows). They are reactive in nature, otherwise negative scan.

Positron emission tomography-computed tomography (PET-CT) scan demonstrated two right and one left small hypermetabolic nodal lesions in the upper jugulodigastric area (Figure 1). 

A surgical dacryocystorhinostomy was performed. The exploration of lacrimal sac revealed a full sac of tumor with indistinct walls and posterior extension of the tumor to the orbit. Remnants of the tumor in the posterior aspect were not removed totally.

**Figure 2:**
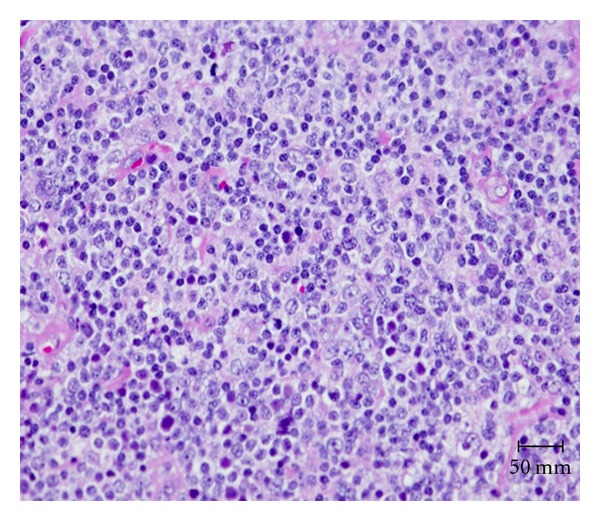
Hematoxylin and Eosin (H&E) stain shows Monotonous lymphoid infiltrate with medium size nuclei.

**Figure 3:**
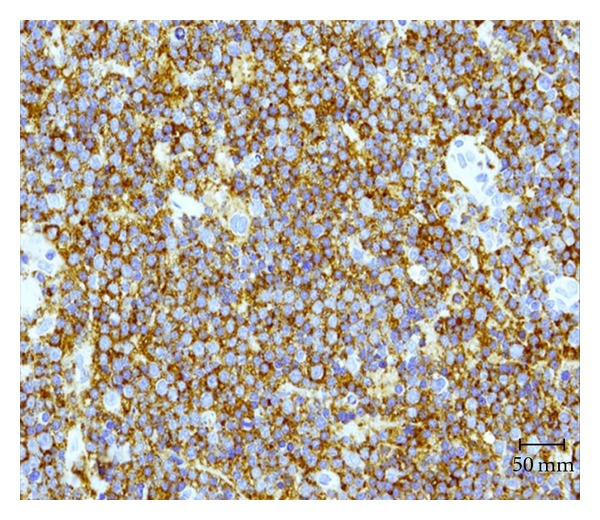
CD 20 immunoreactive lymphoid infiltrate—MALT B-cell lymphoma. Clonality confirmed by immunoglobulin heavy (IGH) gene rearrangement studies favored B-cell extranodal marginal zone lymphoma (MALT type).

Immunohistochemistry revealed atypical lymphoid infiltrate consistent with B-cell non-Hodgkin lymphoma (Figure 2). Clonality confirmed by immunoglobulin heavy (IGH) gene rearrangement studies favored B-cell extranodal marginal zone lymphoma (MALT type) (Figure 3).

Combined chemotherapy and radiotherapy was planned after surgery to eradicate the remnants of the tumor in the posterior aspect, but the family refused the treatment.

The patient was referred to Gastroenterology clinic for looking to *H. pylori* infection of the stomach. He had no gastrointestinal symptoms. Esophagogastroduodenoscopy was performed. Antral nodularity was discovered. Campylobacter-like organism (CLO) test was positive. Multiple biopsies from the antrum and body of the stomach demonstrated chronic active gastritis with heavy helicobacter pylori organisms; no gastric metaplasia, dysplasia, or malignancy. The patient received both Amoxicillin 500 mg q12 hour and Clarithromycin 250 mg q12 hour for two weeks in addition to Esmoprazole 20 mg q12 hour for two months. Two following post therapy, stool antigen for *H. pylori* was negative. Six months after therapy, stool antigen for *helicobacter pylori* was also negative.

12 months later the lacrimal gland tumor was no longer visible on MRI, and the patent had no tearing or ptosis. His eye movement was full, and vision was 20/20 bilaterally without correction.

No extraorbital manifestation of the MALT lymphoma occurred during the subsequent follow-up period of four years.

## 3. Discussion

Mucosa-associated lymphoid tissue (MALT) lymphomas are composed of morphologically heterogeneous small B cells and sometimes plasma cells growing in marginal zones and interfollicular areas [5]. They predominantly occur in older patients and are extremely rare in children and young adults. Among 2,703 children and adolescents registered into the prospective multicenter NHL-BFM treatment studies since 1986, only 4 patients (0.1%) displayed features of MALT lymphoma [6]. MALT lymphoma in the pediatric population has been mainly described in patients with human immunodeficiency virus infection [7], but some isolated case reports of patients with MALT lymphoma and without evidence of immunodeficiency have also been published [8]. MALT lymphoma of ocular adnexa arises in the orbit, conjunctiva, eyelids, and lacrimal gland.

The gastrointestinal tract is the most frequent site of extranodal lymphoma, and the stomach is involved in up to two-thirds of these cases, accounting for 30%–45% of all extranodal lymphoma [9].

The relationship between *H. pylori *and gastric MALT lymphoma is well established and also is thought to play a role in the pathogenesis of conjunctival MALT lymphoma and other nongastric lymphoma [10]. 

The pathogenesis of *H. pylori* and lacrimal MALT lymphoma is not clear. 

MALT lymphomas arise at sites of chronic antigenic stimulation [11] due to autoimmunity (e.g., Hashimoto thyroiditis) or infections (such as *H. pylori*-associated chronic gastritis) [12]. The chronic antigen stimulation hypothesis holds that a specific infectious agent initiates a reactive lymphoid infiltrate in the normally sterile ocular adnexal tissues. This ultimately leads to a B-cell clonal expansion and proliferation. At this stage, genetic alterations and microenvironment may sustain the growth independent of the infectious agent.

There is a linkage between infection with *H. pylori* and chronic atrophic gastritis, an inflammatory precursor of gastric adenocarcinoma [13]. Additionally, regression of gastric MALT lymphoma after eradication of *H. pylori* infection with antibiotics is also consistent with this postulate [14]. Recently, similar regression of disease has been reported in ocular adnexal MALT lymphoma after treatment with antibiotics against Chlamydia psittaci [15]. 

Few data is found on the effect of possible eradication of *H. pylori *infection on MALT lymphoma of lacrimal gland. Ferreri et al. [15] demonstrate the rates of *H. pylori *gastric infection in patients with ocular adnexal lymphoma. Out of the 31 patients with ocular adnexal MALT lymphoma, 10 (32%) have gastric *H. pylori*, and 4 are treated with *H. pylori-*eradicating antibiotics exclusively [15]. The ocular adnexal MALT lymphoma in these patients shows no response; six receive *H. pylori*-eradicating antibiotics concurrently with other therapies (doxycycline, rituximab, or orbit irradiation) achieving lymphoma regression in all cases.

Gruenberger et al. [16] demonstrate case series of 45 patients with MALT lymphoma of the ocular adnexa; 15/39 (38%) have *H. pylori* infection.

Decaudin et al. [17] have examined a large series of gastric biopsies for *H. pylori* in three cohorts of patients: (A) patients with ocular adnexal lymphoma (OAL) (*n* = 83); (B) patients with extragastric and extraophthalmologic lymphoma (*n* = 101); and (C) patients with no clinical evidence of lymphoma (controls; *n* = 156). Gastric *H. pylori* infection was investigated by means of histopathological analysis and *H. pylori*-specific polymerase chain reaction (PCR) assay on gastric biopsy tissue samples and was demonstrated in 45%, 25%, and 12% of patients in groups (A), (B), and (C), respectively. That is, a significant association between gastric *H. pylori* infection and OAL could be demonstrated in this study, with this association being greatest for EMZBL group of OAL. This association with gastric *H. pylori* infection was not seen in other subtypes of OAL. Decaudin et al. propose that gastric *H. pylori* infection results in the initial malignant transformation of the lymphocytes and that these neoplastic B cells subsequently migrate to the ocular adnexa, here forming “ectopic lymphoma.” The migration process is suggested to be as a result of cellular homing mechanisms with chemotactic agents being released by localised chronic inflammation in the ocular adnexal tissues.

Our patient and Decaudin et al. data [17] would suggest that lacrimal MALT lymphoma patients should be examined for gastric *H. pylori* infection following lymphoma diagnosis and that, if positive, antibacterial treatment for *H. pylori* could possibly result in the disappearance of an EMZBL or MALT lymphoma of the lacrimal gland if treated early enough, as seen in gastric MALT lymphomas.

While chemotherapy and/or radiotherapy is the role for cure in adult MALT lymphoma, childhood MALT lymphoma tends to follow an indolent course, and remission could be attained with eradication of *H. pylori* [18]. 

Our case with lacrimal MALT lymphoma after eradication of *H. pylori* infection has prolonged remission for four years without chemotherapy or radiotherapy, similar to Berrebi et al. [19] in which reports one case of MALT lymphoma arising in a minor salivary gland in a patient with gastric *H. pylori *infection, which resolves following treatment of the gastric infection.

## 4. Conclusion

We believe that there is possible new role for *H. Pylori* in the development of MALT lymphoma of the lacrimal gland because *H. pylori* eradication allows prolonged remission of lacrimal MALT lymphoma for four years without chemotherapy or radiotherapy. We suggest that the examination for gastric *H. pylori* infection can be one the of treatment modalities of MALT lymphoma of the lacrimal gland.
